# The Therapeutic Potential of Neuronal K-Cl Co-Transporter KCC2 in Huntington’s Disease and Its Comorbidities

**DOI:** 10.3390/ijms21239142

**Published:** 2020-11-30

**Authors:** Katie Andrews, Sunday Solomon Josiah, Jinwei Zhang

**Affiliations:** Institute of Biomedical and Clinical Sciences, Medical School, College of Medicine and Health, University of Exeter, Hatherly Laboratories, Exeter EX4 4PS, UK; ka387@exeter.ac.uk (K.A.); josiahsos2014@gmail.com (S.S.J.)

**Keywords:** GABAergic, Na^+^-K^+^-2Cl^−^ cotransporter 1 (NKCC1), K^+^-2Cl^−^ cotransporter 2 (KCC2), Chloride (Cl^−^) homeostasis, Huntington’s disease, sleep disorders

## Abstract

Intracellular chloride levels in the brain are regulated primarily through the opposing effects of two cation-chloride co-transporters (CCCs), namely K^+^-Cl^−^ co-transporter-2 (KCC2) and Na^+^-K^+^-Cl^−^ co-transporter-1 (NKCC1). These CCCs are differentially expressed throughout the course of development, thereby determining the excitatory-to-inhibitory γ-aminobutyric acid (GABA) switch. GABAergic excitation (depolarisation) is important in controlling the healthy development of the nervous system; as the brain matures, GABAergic inhibition (hyperpolarisation) prevails. This developmental switch in excitability is important, as uncontrolled regulation of neuronal excitability can have implications for health. Huntington’s disease (HD) is an example of a genetic disorder whereby the expression levels of KCC2 are abnormal due to mutant protein interactions. Although HD is primarily considered a motor disease, many other clinical manifestations exist; these often present in advance of any movement abnormalities. Cognitive change, in addition to sleep disorders, is prevalent in the HD population; the effect of uncontrolled KCC2 function on cognition and sleep has also been explored. Several mechanisms by which KCC2 expression is reduced have been proposed recently, thereby suggesting extensive investigation of KCC2 as a possible therapeutic target for the development of pharmacological compounds that can effectively treat HD co-morbidities. Hence, this review summarizes the role of KCC2 in the healthy and HD brain, and highlights recent advances that attest to KCC2 as a strong research and therapeutic target candidate.

## 1. Introduction

**Figure 1:**
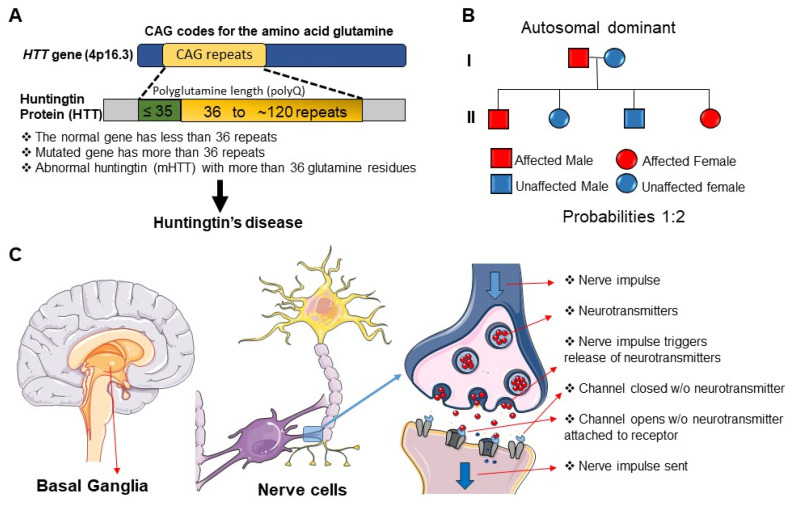
The basic neurobiology of Huntington’s Disease. (**A**) Huntington’s disease (HD) is caused by mutation in the *HTT* gene, located on chromosome 4. The *HTT* gene consists of CAG (cytosine–adenine–guanine) trinucleotide repeats at the 5′ end, which encode glutamine. In healthy individuals, the CAG sequence is repeated 10–35 times, while in HD, the CAG sequence is repeated more than 36 times. The mutated *HTT* gene (*mHTT*) causes the production of abnormal huntingtin (Htt) protein. Htt, with an unusually long polyglutamine sequence, is cut into smaller fragments that will accumulate in neurons; given their toxic nature, cell damage occurs. (**B**) Huntington’s disease (HD) is an autosomal dominant disorder; individuals homozygous (HH) or heterozygous (Hh) for the dominant allele will develop HD. In this example, we have an affected male (Hh) and an unaffected female (hh); therefore, the probability that their offspring will develop HD is 50% (2 in 4). (**C**) Neural cell damage, and subsequent neural cell death in the basal ganglia contributes to the observed symptoms of Huntington’s disease (HD). Electrical signals (nerve impulses) are the basis for communication in the brain; these signals are quickly transmitted from cell to cell via chemical signals known as neurotransmitters. Once generated, a nerve impulse will travel along the length of the axon until it reaches the synaptic knob. At this point, the release of neurotransmitters is triggered; these neurotransmitters will cross the synaptic cleft and bind to complementary receptors on the post synaptic cell. The signal can then be sent along the axon of this second neuron. In HD, basal ganglia structures are smaller than those observed in healthy individuals; this shrinkage is due to death of the striatum. As a result of striatal cell death, the internal globus pallidus (IGP) can only receive a decreased concentration of neurotransmitters.

Huntington’s disease (HD) is an autosomal dominant disorder, caused by CAG trinucleotide repeat expansion of the gene encoding huntingtin (HTT) [1]. While the disease displays complete penetrance, significant interindividual variation in age of disease onset is observed [2]. CAG repeat length only partially explains this variance [3], additional influences include other genetic modifiers [4,5,6], such as epigenetics [7] and environmental aggressors [2,8] (also see Figure 1A,B).

HD is predominantly characterised by progressive motor incoordination; patients tend to experience involuntary muscle contractions and fine motor control defects [9,10]. HD literature demonstrates the progressive death of both striatal and cortical neurons [11,12,13,14]; however, γ-aminobutyric acid (GABA)-ergic projecting neurons of the dorsal striatum are at the highest risk of destruction [2] (Figure 1C). Since striatal neurons play an important role in motor planning and voluntary movement, striatal damage may be responsible for the movement defects experienced [15]. Destruction of the striatum may also exacerbate non-motor symptoms due to its involvement in cognition and behaviour [16].

Patients tend to have several other clinical manifestations such as learning and memory deficits [17,18,19], as well as changes in their sleep architecture [2,10,20,21]. Approximately 90% of HD patients have sleep disturbances; these are often first observed in the premanifest stage of disease, coinciding with the emergence of early cognitive changes [17,18,19,20,21]. Although cognitive and behavioural changes are thought to present in advance of motor symptoms (often several years prior) [19,22,23,24], clinical diagnosis is still largely centred around movement abnormalities [20,25], at a time when striatal cell death is extensive [11,12,13]. However, human brain post-mortem studies have shown that patients with both clinical manifestations (such as behavioural changes) and genetic confirmation of HD have limited neural cell loss [26]. There is evidence of marked variation in the extent and therefore severity of neuropathological changes [26] (also see Figure 1B). This provides evidence that the disease process involves synaptic dysfunction in advance of cell death [27,28]; it is important to appreciate that clinical abnormalities may also present in advance of anatomical changes [26]. The recognition of these cognitive and behavioural abnormalities may better inform early clinical diagnosis of the disease and treatment of these coexisting disorders, thereby improving patient quality of life. In addition, aberrant synaptic signalling may represent a novel therapeutic target.

GABA signalling is crucial in both motor and behavioural control; GABAergic neurotransmission is altered in HD [2,29]. To better understand GABAergic activity, we need to consider both K^+^-2Cl^−^ cotransporter 2 (KCC2) expression and the maintenance of neuronal intracellular chloride (Cl^−^) concentration ([Cl^−^]_i_)_._ Cl^−^ is an important anion involved in the regulation of cell volume [30], proliferation, and apoptosis [31]. Cl^−^ has a further role in determining membrane potential and the firing of action potentials [32]. Extracellular [Cl^−^] tends to be fixed, while [Cl^−^]_i_ is more variable [33]. The presence of chloride-cation cotransporters (CCCs) is central to determining [Cl^−^]_i_ [34]. Such transporters are responsible for the bidirectional movement of Cl^−^, and its function is determined by the direction of flux [31].

GABA is the main inhibitory neurotransmitter in the brain [35]. GABA binds GABA type A receptors (GABA_A_R); these receptors are ligand-gated anion channels central to the control of Cl^−^ movement [35]. Noteworthily, GABA_A_ receptors are permeable for both Cl^−^ and bicarbonate (HCO_3_^−^); the net effect of GABA therefore also depends on the distribution of bicarbonate [36]. Previous studies have also implicated HCO_3_^−^ in GABA_A_ receptor-mediated depolarization [37,38,39,40,41]. In a recent study, Lombardi et al. [41] suggest that implementation of physiological levels of HCO_3_^−^-conductivity to GABA_A_ receptors enhances the [Cl^−^]_i_ changes over an extensive range of [Cl^−^]_I_; however, this outcome strictly depends on the stability of the HCO_3_^−^ gradient and the intracellular pH. For in-depth understanding on the relationship between distribution of HCO_3_^−^ and GABA signalling, readers are referred to recent review on the subject [36,42,43,44]. Yet, the reversal potential (when net flow = 0) for GABA (*E*_GABA_) is primarily determined by the reversal potential for Cl^−^; GABAergic signalling is therefore dependent on [Cl^−^]_i_ [45]. Whilst inhibitory GABAergic activity is important for proper central nervous system (CNS) functioning [46], GABA can also induce membrane depolarisation [47]. The excitatory action of GABA is important in the development of the nervous system [35]; its roles include regulating synaptogenesis in addition to supporting neurite outgrowth and the maturation of the neuronal network [2,35]. High [Cl^−^]_i_ produces less negative GABA currents that culminate in depolarisation events (excitation) [48]. Conversely, low [Cl^−^]_i_ leads to hyperpolarisation as a result of more negative *E*_GABA_ values (inhibition) [45]. During development there is a gradual hyperpolarising shift in *E*_GABA_ as a result of decreased [Cl^−^]_i_, which is maintained in the mature mammalian brain [49,50]. Since the healthy brain relies on the proper balance between excitatory and inhibitory inputs, uncontrolled regulation of neuronal excitability can have implications for health [32].

**Figure 2:**
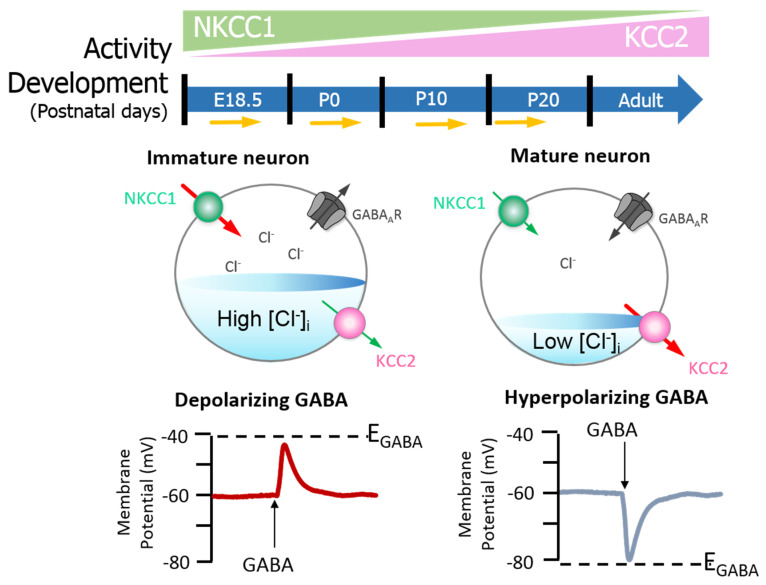
GABA_A_ signalling shifts from depolarizing to hyperpolarising responses are mediated by developmental expression of KCC2 and NKCC1 in the brain (neocortical neurons) of rats. The differential expression of these channels regulates intracellular Cl^−^ concentration ([Cl^−^]_i_) and therefore determines the activity of γ-aminobutyric acid (GABA). Na^+^–K^+^–Cl^−^ cotransporter 1 (NKCC1) pumps Cl^−^ into neurons; its expression is high in the early postnatal period, decreasing as maturation proceeds. The expression pattern for K^+^–2Cl^−^ cotransporter 2 (KCC2), responsible for Cl^−^ efflux, is directly opposite. In the embryonic and early postnatal periods, [Cl^−^]_i_ is high, and so GABAergic signalling is excitatory (depolarising); as maturation occurs, [Cl^−^]_i_ decreases, initiating the development hyperpolarising shift, whereby GABAergic signalling becomes inhibitory. Figure elements were taken and modified from Tillman and Zhang [63].

Neuronal [Cl^−^]_i_ is largely regulated through the activity of Na^+^-K^+^-2Cl^−^ cotransporter 1 (NKCC1) and KCC2 [51,52,53]. NKCC1 pumps Cl^−^ into neurons, while KCC2 is responsible for Cl^−^ efflux [50,53]. Moreover, the extensively studied CCC family member, NKCC1, has numerous physiological obligations [54,55,56] that make it a promising neurological drug target, owing to its importance in GABAergic signalling [50]. Recently, Chew and colleagues [57] determined the cryo-electron microscopy structure of NKCC1 from *Danio rerio*. This extensive study revealed the mechanisms involved in NKCC1 molecular transportation and communication and further provided insights into ion selectivity as well as coupling and translocation; a clearer framework for understanding the physiological functions of NKCC1 in relation to human diseases was also established [57]. Besides, modulation of NKCC1 activity alongside that of KCC2 has been implicated in the development and progression of HD [2,58,59,60]. These CCCs are differentially expressed over the course of development, and so the activity of KCC2 and NKCC1 is not synonymous between immature and mature neurons [50]. In the embryonic and early postnatal period, solute carrier family 12 (SLC12), member A1 (SLC12A1) messenger RNA (mRNA) expression of NKCC1 is high [50]. As maturation proceeds, NKCC1 expression decreases and SLC12, member A5 (SLC12A5) expression of mRNA encoding KCC2 is upregulated: there is a resultant net decrease in [Cl^−^]_i_ [50]. The developmental stimulation of KCC2 and inhibition of NKCC1 expression initiates the switch from excitatory to inhibitory GABA signalling [61]. These evolutionarily conserved transporters (KCC2 and NKCC1) are inclusive among central mediators of ion transport in multicellular organisms, with specific roles in regulating ionic and water homeostasis in mammalian CNS [62], which is essential in determining the polarity of the neurons [63]. Notably, during development, [Cl^−^]_i_ increment is prominent in immature neurons and when activated, they display a depolarising response, which is due to the elevated expression of NKCC1 in comparison with KCC2 [50]. However, during maturation, NKCC1 expression gradually decreases, and KCC2 expression increases, resulting in an opposite expression pattern [50,63] (also see Figure 2). Importantly, the inhibition/stimulation of KCC2/NKCC1 pair via protein phosphorylation is through a regulatory mechanism that works in a reciprocal pattern [53,64] and members of the with-no-lysine kinase (WNKs) family as well as their downstream targets; STE20/SPS1-related proline/alanine rich kinase (SPAK) and oxidative stress response kinase (OSR1) are the most prominent kinases that regulate this process [52,64,65,66,67]. Consequently, impaired ion homeostasis resulting from mutation in the physiological function of some of this transporter pair and/or their upstream regulators may be detrimental and subsequently result in diminished inhibition and augmented network hyperexcitability, which underlies numerous neurological disorders [52,66,68,69,70,71] including HD [58,59,60].

Indeed, loss of KCC2 has implications in disease: KCC2 dysfunction and/or deficiency attenuates Cl^−^ efflux and GABAergic inhibition is therefore impaired [32,46]. When [Cl^−^]_i_ exceeds equilibrium, depolarisation events contribute to the onset of neurological disease [35,46]. Decreased KCC2 expression coupled with increased NKCC1 expression and/or activity has been documented in several pathologies [63,68,69,71], including HD [58,59,60]. In HD, HTT is mutated (mHTT) and acts to alter KCC2 and NKCC1 expressions and activity [58,59] through mechanisms that remain undetermined. Since KCC2 and NKCC1 expressions and functionality are crucial in determining the effects of GABA dysregulated KCC2 and NKCC1 activities [32,46,58,72], subsequent abnormalities in GABAergic signalling are thought to contribute to HD pathogenesis [2]. Hence, this review aims to investigate the possible mechanisms by which KCC2 expression and function is altered in HD. Although HD is primarily characterised by uncoordinated motor activity [9,10], patients have additional, co-existing neurological disorders [17,18,19,20,21]. In view of the aforesaid, the association between altered KCC2 activity, and the comorbidities that present as part of the disease process will also be discussed. For examples, how are NKCC1 and KCC2 expression and activity controlled in the healthy brain? What are the known mechanisms by which KCC2 expression and activity are altered in HD? Additionally, how does altered KCC2 expression and activity contribute to HD comorbidities with a particular focus on cognitive and sleep changes?

## 2. KCC2 Regulation and Function in the Healthy Brain

### Phosphorylation Regulation of KCC2 by Protein Kinase Signalling Pathways

**Figure 3:**
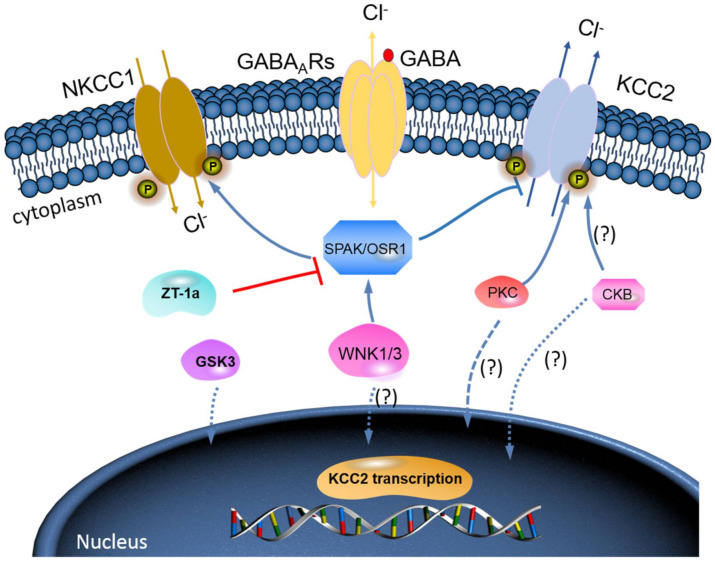
A novel strategy to facilitate neuronal Cl^−^ extrusion and E_GABA_ by coincident NKCC1 inhibition and KCC2 activation by inhibiting the WNK-SPAK/OSR1 kinases. Mammalian neurons that are challenged with multiple neuropsychiatric conditions (such as seizures, neuropathic pain, spasticity, schizophrenia, and others) are usually driven by hyperexcitable circuits, intraneuronal Cl^−^ ([Cl^−^]_i_) levels are elevated due to increased NKCC1 activity, and/or decreased KCC2 activity, promoting GABA_A_R-mediated membrane depolarization and excitation. In healthy mature neurons, [Cl^−^]_i_ is low due to the opposite activity profile of the CCCs, promoting GABA_A_R-mediated hyperpolarization, which is critical for the proper balance of excitation–inhibition in neuronal circuits. WNK-SPAK/OSR1 inhibition, via the coincident effects of NKCC1 inhibition and KCC2 activation (the main Cl^−^ extrusion mechanism in neurons), might be a potent way of facilitating neuronal Cl^−^ extrusion to restore ionic inhibition in diseases that are characterized by disordered Cl^−^ homeostasis and GABA disinhibition. ZT-1a, a novel molecular compound, can specifically inhibit SPAK signalling pathway, thus interfering SPAK regulation of GABA signalling via NKCC1 inhibition and KCC2 activation [86]. Activation of protein kinase C (PKC) and brain-type creatine kinase (CKB) are likely to increase KCC2 cell surface expression, but the mechanisms involved are still unclear.

The WNK-SPAK/OSR1 phosphorylate threonine residues 906 and 1007 (T906/T1007) and subsequently downregulate KCC2 mRNA gene expression; thus, a decline in its physiological function is observed [73,74]. The phosphorylation of these residues is highest in the early postnatal period, with a gradual decrease throughout development [73,74]; WNK1 activity is at its most reduced level in mature neurons [64,74]. KCC2-T906/T1007 phosphorylation has been shown to decrease by approximately 95% between embryonic day 18.5 (E18.5) and adulthood in mice [75]. This decrease in threonine phosphorylation may contribute to the developmental onset of KCC2 function [73,74], thereby facilitating the upregulation of Cl^−^ extrusion from mature neurons resulting in the hyperpolarising *E*_GABA_ shift [32,76] (also see Figure 3).

Moore et al. [76] demonstrated that preventing KCC2-T906/T1007 phosphorylation in vivo (assessed in knock-in mice), via threonine to alanine mutation, accelerates the onset of KCC2 function in the postnatal period. In the study, *E*_GABA_ values were found to be hyperpolarised across neuronal development (patch-clamp experiment; cultured hippocampal knock-in mouse neurons), i.e., in preventing the phosphorylation of KCC2-T906/T1007, postnatal GABAergic depolarisation activity was largely abolished, thus suggesting that the developmental onset of hyperpolarising synaptic inhibition is dependent on regulated KCC2 phosphorylation [76], and this is further supported by other studies [73,74]. Therefore, potentiating KCC2 function to rescue delayed *E*_GABA_ shift during development may improve cognitive defects [76]. Serine 940 (S940) is another key phosphorylation site in the regulation of KCC2 activity, phosphorylation of S940 is controlled by protein kinase C (PKC) [77,78]. S940 phosphorylation leads to decreased KCC2 internalisation and subsequent increased Cl^−^ extrusion [61], thereby increasing KCC2 function [61,77]. In this regard, Moore et al. [76] further suggest that S940 can be experimentally mutated to alanine (S940A) to prevent its phosphorylation to facilitate [Cl^−^] increment. Briefly, they demonstrated that developmental *E*_GABA_ shift is delayed in S940A neurons compared to wildtype (WT) controls, thereby suggesting that phospho-regulation of KCC2-S940 may be involved in defining the developmental onset of GABAergic inhibition [76].

Furthermore, brain-type creatine kinase (CKB) also plays a vital role in regulating cellular energy homeostasis via ATP-dependent phosphorylated catalysis of creatine into phosphocreatine, thereby establishing a readily available ATP-buffering system [79]. Notably, ATP is involved in the activation of Na^+^-K^+^-ATPase, which serves as a driving force for KCC activation; hence, it is expected that ATP should enhance the function of KCC2 [80]. Interestingly, some reports have affirmed an ATP-induced KCC activation [27,28]. Aside from potentially providing ATP, Hemmer et al. [81] hypothesize that CKB might phosphorylate KCC2 to change its function, because CKB possesses autophosphorylation activity. However, the implication of the interaction between KCC2 and CKB in relation to their physiological functions and how intracellular ATP concentrations might contribute to KCC2 function is still elusive [80]. More importantly, however, the fact that WNK-SPAK/OSR1 kinase complex is known to phosphorylate and inhibit KCC2 or stimulate NKCC1 [52,64,65,66,67] is already established. Thus, molecular compounds that can block WNK-SPAK/OSR1 signalling pathway will result in activating KCC2 and inhibiting NKCC1 activities. The manipulation of the interaction between CKB and KCC2 activities could be a substitute mechanism to achieve KCC2 activation [80,82]. In fact, the interaction between CKB and KCC2 expression/activity has been implicated in the modulation of GABA_A_R-mediated signalling [2,59]. Furthermore, previous reports have demonstrated that enhancement of CKB activity may facilitate the activation of KCC2 function [82,83,84,85]. In HD, reduced expression and activity of CKB is associated with motor deficits and hearing impairment [83,84]. By and large, the enhancement of CKB activity prior to its interaction with KCC2 activates its function resulting from inhibited phosphorylation of the WNK-SPAK/OSR1 signalling pathway may be a hypothesis worthy of intensive investigations (Figure 3). Hence, it is worthwhile to further investigate the interaction of KCC2 and CKB and how the interaction can modulate the WNK-SPAK/OSR1 signalling cascades in neurological diseases including HD.

Indeed, phosphorylation status of key regulatory sites on KCC2 determines when the developmental *E*_GABA_ shift occurs, and regulated depolarising GABAergic signalling (largely in the early postnatal period) is necessary for normal cognitive and behavioural development [76]. Since the phosphorylation process is central to KCC2 function, future research should assess whether the phosphorylation status of key KCC2 sites is constant between HD patients and controls. Further to this, it should be established if phosphorylation status changes as HD progresses. If the phosphorylation of key KCC2 sites does not occur as normal in HD gene carriers and patients, investigating how this affects the development hyperpolarising shift in *E*_GABA_ is of concern; this research may provide an explanation for the behavioural and cognitive manifestations observed in HD patients. Investigation into the phosphorylation of KCC2 and the *E*_GABA_ is of increasing interest, especially since it has been suggested that the potentiation of KCC2 function (accelerating hyperpolarising shift) can improve cognitive decline [76].

## 3. KCC2 Regulation and Function in the HD Brain

To start with, dysfunctions in GABAergic inhibitory neural transmission happen in neurological disorders including HD [2,59,72]. KCC2 is a key moderator of inhibitory GABAergic inputs in normal/healthy adult neurons, as its Cl^−^ extruding activity facilitates the hyperpolarizing reversal potential for GABA_A_R Cl^−^ currents and its disruption promotes HD-associated symptoms [2,29,59,76,87]. Certainly, KCC2 interacts with HTT and is downregulated in HD, which contributed to GABAergic excitation and memory deficits in the R6/2 mouse HD model [2,59]. Recently, Dargaei et al. [59] demonstrated that aberrant CCC expression causes a shift in the reversal potential of GABA_A_R-mediated Cl^−^ currents, resulting in excitatory GABA_A_R signalling. In the study, HD transgenic mice (R6/2) have decreased KCC2 and increased NKCC1 activity replicating CCC expression observed in the brains of HD patients [59]. Particularly, decreased expression of KCC2 mRNA protein was more prominent in the cortical and striatal regions coupled with significant reduced expression of KCC2 in the hippocampus of HD brains of R6/2 [59]. Noteworthily, recent works have now potentially indicated that pharmacological enhancement of KCC2 function could reactivate dormant relay circuits in injured mouse and patient brain, leading to functional recovery and the amelioration of neuronal abnormality and disease phenotype associated with mouse and human models of neurological disorders including HD [58,59,60,86,88,89]. Indeed, there is a growing potential for KCC2 as a vital therapeutic target for neurological diseases and subsequent inhibitory input dysfunctions [60].

### 3.1. Mechanisms of Reduced KCC2 Function in HD

Several theories exist as to why KCC2 expression is reduced in the brain of HD patients and some of these theories, in one way or the other, implicate CKB, a KCC2-interacting protein [2,59,84,85]. In a recent mouse model study, Hsu et al. [85] demonstrated that interactions as well as the expression levels of KCC2 and an interacting protein, CKB, are reduced in neurons of R6/2 when compared with WT. In this study, the researchers treated the animals with vehicle as well as drugs that selectively target synaptic or extrasynaptic GABA_A_ receptors (diazepam or gaboxadol) and subsequently used real-time quantitative polymerase chain reaction, western blot, and immunocytochemistry techniques to monitor the GABA_A_R and KCC2 expression levels; they further evaluated the interaction between KCC2 and CKB in primary cortical neurons harvested from WT and R6/2 using immunofluorescence and proximity ligation assays [85]. In conclusion, the results from that study suggested that reduced CKB and KCC2 function occurred in HD neurons, which may diminish the GABA_A_-mediated inhibitory function [85]. Additionally, Dargaei et al. [59] suggested that KCC2 may be appropriated into mHTT inclusions in the hippocampus, which greatly interfere with the transporter’s expression and functionality, and that the possible effect of mHTT on KCC2 function may be due to the interaction between KCC2 and CKB. Noteworthily, decreased CKB expression in mHTT expressing neurons is a significant event in the development and progression of HD, which certainly contributes to the neuronal dysfunction linked with HD [79,83,84]. In addition to that, CKB interacts, phosphorylates, and activates KCC2 expression/function [80,82]; hence, diminished KCC2 function in HD is most likely to occur, which may subsequently reduce GABA_A_-mediated inhibitory function [2]. In view of the aforementioned, Dargaei and co-workers [59] briefly hypothesised that the observed decrease in the hippocampal KCC2 expression of R6/2 mice may result from reduced CKB-mediated phosphorylation and activation of KCC2. Furthermore, decreased KCC2 expression and activity may be a result of the toxic effects of mHTT, as the mutant protein may cause aberrant protein–protein interactions, forming protein aggregates as part of the disease process [59]. Consequently, the KCC2 protein may be sequestered into these mHTT aggregates, thereby reducing KCC2 functionality in the brain [59].

Both loss-of-function and gain-of-function mHTT effects exist, and loss-of-function effects may be responsible for triggering disease pathogenesis In addition, these effects may produce the neurological characteristics of HD [3]. Gain-of-function effects, on the other hand, may drive disease progression, and current strategies for the treatment of HD often include HTT expression knockdown; these techniques are not specific, and therefore both mutant and WT HTT expression is targeted [3]. Since WT HTT has many functional roles in the CNS, these approaches may trigger unwanted outcomes such as those observed in HD; unfortunately, given HTT knockdown techniques may produce unwanted effects [3]. Hence, further research could aim to refine these techniques to be more directed, or probably identify other targets, such as CKB to increase KCC2 expression and functionality in the HD brain. Further investigations should also seek to replicate these findings. Moreover, it would be interesting to determine if the loss-of-functions effects of mHTT act to trigger disease onset independent of CAG repeat length. 

Other mechanisms involve WT HTT, which has many functional roles [3,20,90,91]. HTT is important in embryonic development with a role in neurogenesis; HTT knockout mice display embryonic lethality [92]. HTT is also an important protein for the control of vesicle transport and gene transcription [3,20], as WT HTT interacts with transcription activators and repressors [90,91]. In HD, it is suggested that mHTT could cause abnormal interactions with transcriptional machinery, thereby contributing to reduced (or aberrant) KCC2 and GABA_A_R subunit expression [2]. Two RE1/NRSE (repressor element 1/neuron restrictive silencer element) sites flank the transcription start site of the KCC2 gene [71]. WT HTT ensures the REST/NRSF (RE1 silencing transcription factor/neuron restrictive silencer factor) complex is maintained in the cytoplasm; in this state, the complex is unable to bind RE1/NRSE, permitting gene transcription [91]. mHTT, however, inhibits the transcription of genes containing NRSF, reducing KCC2 expression [91] (also see Figure 3). Investigations into the significance of REST and RE1 have yielded results which may inform the development of novel therapeutics [71]. For example, REST-dual RE1 interaction may represent a novel mechanism for the upregulation of KCC2, thereby promoting the GABAergic switch from excitatory to inhibitory action [71]. This study also revealed how REST inhibition may accelerate the developmental Cl^−^ shift, while REST overexpression slows the hyperpolarising *E*_GABA_ shift [71]. This may have applications in improving the cognition of HD patients. As discussed earlier, further research should establish the role of the *E*_GABA_ shift in the cognitive and behavioural manifestations of the disease, therefore making REST a potential therapeutic target.

Additionally, many transcription factor-binding sites have been characterised in the SLC12A5 gene [93,94]. For example, the transcription factor, early growth response 4 (Egr4), is enriched in neurons and has been identified as a key regulator in the control of KCC2 expression [93]. Erg4 mediates brain-derived neurotrophic factor (BDNF)-dependent transcription of KCC2 in immature neurons [95]. BDNF, whose expression and activity are altered in HD populations, is important in the survival of striatal neurons [91]. The aforementioned hypothesis was further supported by the findings of Yeo et al. [71] that demonstrated that KCC2 expression may be potentiated by the application of BDNF. In another rat model study, Zhang et al. [96] demonstrated that microinjection of BDNF (1 μg/μL) into the nucleus raphe magnus (NRM) region of the brain significantly inhibited the expression of KCC2 protein in the brainstem of injected rats when compared with control (non-injected) rats. Furthermore, BDNF have been suggested as a strong candidate responsible for downregulation of KCC2 expression in hippocampal cells [97,98]. Interestingly, both BDNF and inhibition of KCC2 produce similar effects in inverting inhibitory GABA synaptic currents in neurons cells, thereby instigating the cellular mechanisms for impaired GABA inhibitory function [96,99]. Previous studies that provide more direct supporting evidence for BDNF ability to decrease KCC2 expression as the signalling mechanism for loss of GABA inhibition do exist [96,100,101,102]. Hence, impairment of the BDNF-KCC2-GABA signalling cascade may promote neurological dysfunctions [96] including HD [91,95]. These findings may provide a means for increasing KCC2 expression in HD, and future study should continue to investigate the association. Similarly, establishing how the activity of Erg4 can be manipulated in order to control and potentially enhance KCC2 expression in the HD brain may be of interest as a therapeutic target.

WT HTT has a further role in synaptic connectivity—it is specifically important in the formation and maintenance of cortical and striatal excitatory synapses; silencing HTT in the developing mouse cortex leads to an increase in excitatory synapse formation [3,103]. Li and Li [104] showed that the altered communication between mHTT and HTT interactors promotes aberrant synaptic transmission in HD. This has significant consequences for patients, since synaptic dysfunction is thought to underlie the mechanisms by which cognitive and behavioural changes manifest [27,28].

Not only is KCC2 expression altered in HD, but NKCC1 expression is also abnormally increased [2,58,60]. In fact, there are several reports alluding that enhanced NKCC1 activity may contribute to the pathogenesis of HD [2,58,59,60]. In a recent mice and human study, Hsu and co-workers [58] demonstrated that NKCC1 mRNA expression increased in the striatum of R6/2 and Hdh^150Q/7Q^ transgenic HD mice and caudate nucleus of HD patients. Furthermore, inhibition of NKCC1 with bumetanide and adeno-associated viral vectors (AAVs) salvaged the motor deficits of R6/2 mice, thereby suggesting NKCC1 as possible therapeutic target for the potential salvage of motor dysfunction in patients with HD [58]. Indeed, increases in NKCC1 expression are seen to accompany reductions in KCC2 expression; this phenomenon is thought to be as a result of KCC2 reversion to its immature GABAergic phenotype, NKCC1 [2,59,60]. Additionally, upregulation of NKCC1 expression leads to a higher [Cl^−^]_I_, since it allows an influx of Cl^−^ and thus when GABA is stimulated, causes an excitatory response [105]. Increased NKCC1 expression in disease may also be as a result of the toxic secondary effects of mHTT; for instance, mHTT reduces BDNF expression by impairing its gene transcription [59]; mHTT is also thought to be involved in the inhibition of BDNF release and transport [106]. WT HTT sustains the production of cortically derived BDNF, which regulates NKCC1 expression; hence, reduced KCC2 expression and functionality, coupled with increased NKCC1 activity, leads to the disruption of [Cl^−^]_i_ followed by the reversal of *E*_GABA_ [59]. Indeed, excitatory GABAergic signalling promotes disease states, as is the case in HD [2,59,72]. Furthermore, the balance between GABAergic inhibition and excitation is important in processes such as circadian rhythmicity and sleep [107]; this is particularly pertinent for HD patients. The mechanism behind how KCC2 reverts back to its immature phenotype should be established. Similarly, research should further explore mHTT as a therapeutic target.

### 3.2. Sleep Disorders in Huntington’s Disease

Changes in the sleep architecture of HD patients were first described in 2005 [108]. Since then, extensive research has sought to explain the underlying mechanisms for the pathogenesis of such disorders [25,60,72]. It is difficult to accurately measure circadian behaviour (e.g., any changes in rhythmicity) in humans; this is largely due to the fact the environment in which we live varies significantly and may act to disguise endogenous rhythms [109]. However, the sleep alterations seen in HD seem to appear in the premanifest stage and become increasingly worse as the disease progresses [17,18,19,20,21]. One study, for example, assessed the association between circadian blood pressure (BP) changes and sleep quality in HD patients compared to controls (38 HD patients: 23 premanifest; 15 early stage HD and 38 age- and sex-matched controls); based on percentage change classification in day/night time BP, subjects with decrease relative to daytime BP (nocturnal dippers) were ≥10%, while the non-dippers (BP decrease relative to night-time) were <10%. Overall, HD patients were significantly (*p* = 0.001) more likely to experience non-dipping and increased daytime sleepiness compared to controls, both of which indicate poorer sleep quality [25], and these may be implicated in poor cognitive performances [110,111]. Although this study used an objective measure to assess sleep (BP), which has an advantage over other methods such as actimetry (an indirect measure of sleep), the sample size used was relatively small. In view of that, patients were instructed to self-report their sleep quality—this is a subjective measure and therefore open to bias; the use of other techniques such as polysomnography may have yielded differing results. Moreover, six of the participants were under antidepressant prescription (medication was included on the basis that BP changes, if present, remained constant) [25]; such medication may interfere with sleep architecture. For example, drugs with activating effects (e.g., fluoxetine) may cause sleep disturbances [112]. Contrastingly, antidepressants with more sedative-like properties (e.g., doxepin) will act to improve sleep in the short-term but may cause sleep architecture changes with prolonged ingestion [112]. Additional research should therefore seek to replicate these findings, controlling for the outlined confounders.

Evidence of worsened sleep quality in HD patients is further supported by Lazar et al. [113], who demonstrated that non-HD gene carrier patients significantly had better overall sleep quality compared to HD gene carriers, therefore suggesting that sleep quality decreases with disease progression [113]. Frequent nocturnal awakenings and delayed sleep onset in early-stage HD patients have been reported by Goodman et al. [10], who used polysomnography (to assess sleep directly) and actigraphy techniques. These methods are potentially less reliable, since they are dependent upon movement, and HD is predominantly a motor disease [10]. Even though the polysomnography data seemed to support the actigraphy findings, the reliability of the latter suggests that methodological triangulation should be employed. Again, some of the study participants were under antidepressant prescription, which may act as a confounding factor [10]. The seemingly wide use of antidepressants in patients [10,25] further demonstrates the burden of HD comorbidities. Another study found sleep onset and wake-up times to be delayed in HD patients [114]. This study also implicates hippocampal changes, as discussed earlier [59], in the disease process; HD patients had worsened sleep quality, which was seen to be associated with decreased cognitive performance [114]. It should be noted, however, that this study used questionnaires to investigate sleep rather than an objective measure such as polysomnography; additionally, the employed sample size was relatively small [114]. It is vital to either assess sleep through direct, objective measures, or confirm subjective findings with objective research methodologies [115].

Indeed, difficulty in falling asleep is among the most prevalent of symptoms in HD [116]. Jha et al. [116] report that sleep changes are dependent on disease duration and severity but did not find a significant correlation between CAG repeat length and sleep disturbances. This is an interesting point, since severity of disease is normally defined by the length of CAG repeats [2]. Future research should investigate whether an association does in fact exist. A study in HD sheep supports the notion that sleep changes are amongst the earliest symptoms of the HD, and not as a result of disease progression [109]. A further study shows early cognitive changes begin to emerge in the premanifest stage of disease [19], the same time at which sleep changes are observed [17,18,19,20,21], thus supporting this point. Cognitive decline may present up to 15 years prior to the emergence of motor symptoms [117]. Other studies, however, report that only select cognitive measures show accelerated decline [118,119], and another study that employed ambulatory electroencephalogram (EEG) recordings demonstrated less compelling evidence [120]. The studies discussed in this section show that HD patients experience excessive daytime sleepiness, delayed wake-up times, nocturnal awakenings, and sleep fragmentation [10,25]. These are all indicative of worsened sleep quality, and since sleep is central to proper human functioning, may exacerbate cognitive deficits [110]. If we can effectively treat sleep disorders when they first manifest, we may also improve the cognitive and learning and memory deficits patients also report. However, selecting the most appropriate animal model for the investigation of human pathologies is imperative. R6/2 mice are the best characterised rodent model; they do however have limitations: they carry only a portion of the HD gene and have a shorter lifespan and different neuroanatomical organisation [109]. Whether data collected through the use of murine models is entirely translational to the human HD population is therefore under debate [109]. Given sheep carry the full HD gene (with relevant CAG repeat length) [109], they may represent a useful animal model for the investigation of the mechanisms underlying these circadian changes and how these changes may influence disease progression. While sheep present other challenges as a disease model (size, maturation period etc.), the ovine model could be used to confirm or disprove current findings.

### 3.3. Sleep Disorders Treatment

Co-existing morbidities in HD populations often cause serious distress to patients and their relatives [121]. Studies have found that poor sleep quality is associated with irritability and depression, independent of each other [114,122]; this is true of HD patients also [10,113]. As discussed, sleep quality is thought to worsen throughout the progression of HD [113], possibly exacerbating other clinical manifestations such as cognitive decline [123] and learning and memory abilities [114,122]. Promisingly, mouse studies have shown that cognitive deficits can be restored through the pharmacological manipulation of sleep; such intervention may act to improve sleep quality and wakefulness, thereby improving cognition [124]. Although a cure for HD does not exist, the neuropsychiatric symptoms that present alongside the disease are largely treatable [121]. Anderson et al. [121], advise the use of randomised control trials to establish the best treatment options. Nonetheless, clinical statements are currently available to guide the management of HD comorbidities based on the treatment options previously developed for use in non-HD populations [3,121]. For example, GABA is already used in the treatment of sleep disorders [125]. Further research could establish whether GABA or CCC agonists/antagonists can be used as therapeutic agents for sleep disorders in HD patients. 

### 3.4. Hypothalamic Changes in the HD: Implications for Sleep and Circadian Rhythmicity

Very few studies have sought to assess hypothalamic changes in HD patients; this is partly due to shortages in tissue availability from this region, but is also as a result of differing definitions regarding the boundaries of the hypothalamus and its nuclei [20]. Despite this, hypothalamic dysfunction has been evidenced in the early stages of HD [126]. Early studies, using magnetic resonance imaging techniques showed loss of grey matter in the HD hypothalamus [127,128]. At this time, Petersen and Gabery [20] also observed atrophy in the hypothalamus of both R6/2 transgenic mice and HD patients. More recently, Politis et al. [126] have evidenced hypothalamic involvement in HD by demonstrating the loss and/or dysfunction of dopamine receptors in premanifest HD gene carriers and symptomatic patients. This hypothalamic dysfunction has been implicated in poorer sleep quality of HD patients [25], and may also cause autonomic dysfunction [20,25,129].

It is thought that HD hypothalamic changes occur independently of striatal alterations; this could therefore explain differences in the severity and extent of the comorbidities observed [126]. Furthermore, since HD diagnosis is centred around the presence of motor abnormalities, research largely focusses on explaining the underlying mechanisms for these changes; investigation of other clinical manifestations, which are now thought to present prior to motor impairment, is less extensive. The recognition and investigation of HD hypothalamic changes are of critical importance for the study of sleep disorders in HD patients. This is because the suprachiasmatic nucleus (SCN), considered the pacemaker for circadian rhythmicity, is located in the anterior hypothalamus [130,131]. The SCN is both the brain’s clock and the brain’s calendar [132]. The ability of the SCN to respond to changing seasonality is critically important in maintaining several biological processes to which neurotransmission and sleep are central [132,133,134].

### 3.5. KCC2 and GABA Involvement 

The mechanisms underlying circadian rhythmicity disruption and sleep disturbances remain unclear [120]. There is, however, evidence that mouse and rat transgenic models of HD may be able to replicate the sleep disorders reported in HD patients, thereby providing an important means for understanding these mechanisms [120]. GABAergic signalling, as well as KCC2 expression and functionality, may play an underlying role in these mechanisms. GABA is the primary neurotransmitter in the SCN [135]; it is the only neurotransmitter that is produced and received by SCN neurons [136]. Furthermore, both GABA_A_ and GABA_B_ receptors are present in more than 90% of SCN neurons [131,135,137,138]. GABA_A_R activity controls the ability of the pacemaker to shift state in response to light [131,135,139]. Furthermore, SCN state switching depends on Cl^−^ transport and GABA_A_ signalling [132]; in turn, these are dependent on controlled NKCC1 and KCC2 expression and function [132,140].

Contrary to the notion that GABA is inhibitory in the adult CNS, GABAergic excitation has been observed in subsets of matured SCN neurons of rats during a 24-h cycle and was particularly high during the night phase [105,141]. Experiments using immunohistochemistry techniques have shown regional differences in CCC expression in the SCN [142]; [Cl^−^]_i_ and GABAergic excitation also vary on this basis [143]. Furthermore, the polarity of GABA switches between inhibition and excitation in a time-dependent, cyclic manner [141], again controlled by NKCC1 and KCC2 [105,143]. GABAergic excitation (verses inhibition) is dominant at night [105,141], and more notable excitation activity is observed in the dorsal region of the SCN [105]. A decrease in functional KCC2, coupled with an increase in functional NKCC1, may contribute to the depolarising effect of GABA in SCN neurons as a result of elevated [Cl^−^]_i_ [105,133]. It is important to recall that earlier in this review, we highlighted that KCC2 and NKCC1 are mediators in the regulation of ionic and water homeostasis in mammalian CNS [62], which is essential in determining the polarity of neurons [63] (also refer to Figure 2). Undoubtedly, KCC2/NKCC1 pair is involved in the regulation of [Cl^−^]_i_ of SCN neurons, which in turn influences the response of SCN neurons.

Furthermore, recent reports have suggested that KCC2 play a crucial role in the promotion of GABA inhibition in the SCN neurons [2,132,133,143]. A recent mice model study by Olde Engberink and co-workers [133] demonstrated that KCC2 blockade reverses the polarity of the GABAergic response in the SCN neurons from mice ex vivo. In the study, the KCC2 blocker ML077 instigated an increase in GABAergic excitatory responses in SCN neurons (C57BL/6 mice). Furthermore, 26% of the cells with inhibitory responses to GABA, and half of the neurons which originally did not respond to GABA, became excitatory upon ML077 incubation [133]. A second supporting study used a different KCC2 antagonist (VU0240551) to show the involvement of KCC2 in controlling the [Cl^−^]_i_ of SCN neurons [143]. Conversely, the application of the NKCC1 blocker, bumetanide, has directly opposite effects; bumetanide prevents GABAergic excitation [105]. This provides evidence that NKCC1 activity, responsible for increasing [Cl^−^]_i_, promotes excitatory responses of SCN neurons to GABA [58,144]. 

KCC2 activity is important in maintaining the excitatory/inhibitory ratio under all photoperiod conditions [133]. KCC2 is thought to be modulated by light; its expression is specifically downregulated in compartments of the SCN that receive and process photic input [132]. Though the mechanism(s) that light employs to modulate KCC2 expression is still elusive [132], the change in photic input may result via post-translational regulation of KCC2 expression and/or activity, because extended photoperiod is not likely to facilitate its transcription process, that is, increase KCC2 mRNA gene expression in the SCN neurons [145]. Day to night-time variances in the expression of KCC2 regulators, such as kinases, may, however, be implicated [132]. Similarly, underlying mechanisms for KCC2 downregulation at night may involve transcriptional changes [132]. These are the same mechanisms that were discussed in earlier sections. Since mHTT can affect KCC2 expression in the HD brain [2,59,84], determining phosphorylation activity and transcriptional control of KCC2 within the SCN, in both the healthy and HD brain, would be beneficial to assess whether any changes occur, and if these changes are implicated in HD sleep disorders. 

The link between altered KCC2 expression and altered circadian rhythmicity/sleep disorders in HD patients is not a well-studied area of research. The severity and impact of sleep disturbances in HD patient populations [121] validate why this is a clinically relevant field of investigation; the mechanisms underlying circadian rhythmicity changes urgently need to be understood. Future studies should seek to determine KCC2 and NKCC1 expression levels in the SCN at night and in the daytime, in both healthy and diseased brains. This would be useful in establishing whether changes exist, and if these changes are in fact significant. Further to this, research should investigate whether the downregulation of KCC2, for example, as a result of interactions with mHTT [2,84], or reversion back to NKCC1 [59], contributes to sleep disorders in HD. Additionally, future studies should assess whether the switch between GABAergic inhibition and excitation occurs from day to night as expected, thereby determining whether this represents a possible pharmacological target in HD populations. 

### 3.6. Drug Development for KCC2 Activation

The development of specific and potent NKCC1 inhibitors and KCC2 activators represents a long-sought goal for the treatment of multiple central nervous system (CNS) diseases. As discussed above, WNK1-regulated phosphorylation of KCC2 at Thr906 and Thr1007, by SPAK/OSR1, maintains depolarizing GABA activity in neurons, representing a promising therapeutic drug target for GABAergic inhibition. 

Researchers have made great effects in elaborating inhibitors against WNKs or SPAK/OSR1 to treat hypertension, for example, WNKs kinase inhibitor: WNK463 [146], PP121 [147]; SPAK/OSR1 inhibitors: STOCK1S-14279 and Closantel [148], Rafoxanide [149], Verteporfin [150], STOCK1S-50699, and STOCK2S-26016 [151]. However, none of these compounds have been successfully applied for the treatment of brain disorders due to the low permeability in blood–brain barrier. 

We have now designed and synthesized a new focused chemical library derived from Closantel [148] and Rafoxanide [149], though a “scaffold-hybrid” strategy [51], which led to identification of “ZT-1a” [5-chloro-N-(5-chloro-4-((4-chlorophenyl)(cyano)methyl)-2-methylphenyl)-2-hydroxybenzamide] as a highly selective SPAK inhibitor [86]. ZT-1a provides neuroprotection by directly inhibiting SPAK kinase activity and SPAK-mediated phospho-activation of NKCC1 and phospho-inactivation of KCCs in ischemic brains [86]. Thus, it is promising because ZT-1a may interfere with the SPAK regulation of GABA signalling via NKCC1 and KCC2 through controlling [Cl^−^]_i_ in neurons (Figure 3). We propose that future research should examine whether KCC2 phosphorylation of Thr906/Thr1007 is important for function and pathology in cortical networks through studying HD animal models or further investigating the therapeutic utility and potential of SPAK specific inhibitor ZT-1a treatment.

## 4. Conclusions and Future Prospective

In summary, there is a link between HD and sleep; both sleep questionnaire-based studies and studies using objective measures such as polysomnography and BP monitoring techniques have confirmed this association. The activity of KCC2 is important in the brain, and mHTT may act to alter its expression in HD victims. Furthermore, KCC2 expression in the SCN is central to the control of circadian rhythmicity and sleep. In HD, although most damage is noted in the striatal neurons (most commonly associated with movement), hypothalamic dysfunction also occurs, perhaps before striatal damage. It therefore stands to reason that improper KCC2 expression and/or activity may contribute to sleep disorders affecting a large proportion of HD patients. Furthermore, the controlled expression and function of KCC2 is central to determining sleep–wake cycles. Though knowledge about the underlying mechanisms of altered sleep architecture in the HD brain is yet elusive; it may be possible that KCC2 expression, and its role in determining GABAergic excitation is key to this. Investigating KCC2 as a therapeutic target may therefore lead to the production of pharmacological compounds that can effectively treat HD co-morbidities. Patient quality of life would as a result be enhanced; future research should also assess its applicability to potentially improving disease prognosis.
